# Adaptation and validation of the motivated strategies for learning questionnaire for Dutch pre-vocational students

**DOI:** 10.1186/s40461-025-00188-2

**Published:** 2025-07-03

**Authors:** Bernard Brouwer, Matthijs J. Warrens, Hanke Korpershoek

**Affiliations:** GION education/research, Faculty of Behavioural and Social Sciences, University of Groningen, Grote Rozenstraat 3, Groningen, 9712 TG The Netherlands

**Keywords:** Motivation, Learning strategies, Pre-vocational students, Self-regulated learning, Confirmatory factor analysis

## Abstract

To study self-regulated learning in pre-vocational education, researchers need instruments that have high reliability and validity. This study investigated, for the first time, the factorial validity and reliability of the Motivated Strategies for Learning Questionnaire (MSLQ) for students in pre-vocational education in the Netherlands. The MSLQ was administered to 594 Dutch pre-vocational students. Reliability analyses of the MSLQ sections and subscales were conducted. Furthermore, confirmatory factor analysis was used to test the factor structures of the MSLQ sections, scales and subscales. High reliability was found for both sections of the MSLQ, i.e., the sections Motivation and Learning Strategies, but varying levels of reliability were obtained for the fifteen MSLQ subscales. With regard to factorial validity, a moderate to good model fit for most subscales was found. For some subscales, certain modifications were required to obtain a good model fit. Each of the modifications made could be justified substantially. Furthermore, for two subscales the factor model was exactly identified and factor validity could not be studied. For most MSLQ subscales, evidence was found that the constructs measured with these subscales can be assessed quite well among Dutch pre-vocational students. However, scores based on the complete MSLQ or the two sections should not be used.

**Supplementary Information:**

The online version contains supplementary material available at 10.1186/s40461-025-00188-2.

## Introduction

Decline in student motivation and the high rates of attrition are a real concern in education and have been reported in various countries (Dyrberg and Holmegaard 2019; Young et al. 2018). Motivation to learn is considered indispensable for a successful school career and ultimately for students’ tendency to stay in school (Alexander 2001; Minneart et al. 2017). Educators, policy makers and researchers in the Netherlands have been concerned for years about the decline in achievement and motivational beliefs of students, as well as the decrease in self-regulated learning behaviour (e.g., effort put into schoolwork and strategy use) (Bussemaker 2016; Luyten et al. 2003; Prince 2014; Van der Veen et al. 2005; Van der Veen and Peetsma 2009).

The decline in self-regulated learning behaviour (Peetsma and Van der Veen 2013; Van der Veen and Peetsma 2009) is expected to be more extensive at the lower levels of secondary education compared to higher levels of secondary education (Dutch Inspectorate of Education 2005). In the Netherlands, these lower levels are the pre-vocational tracks, and the higher levels are senior general and pre-university education, both preparing for higher education. The Dutch Inspectorate repeatedly urges schools to put more emphasis on ‘higher-order-thinking skills’, like evaluation and reflection (Dutch Inspectorate of Education 2021). Dropout rates tend to peak in the first year after the transition from pre-vocational schools to vocational education (Alexander 2001; Elffers 2012; Severiens and Verstegen 2007). At vocational schools, students have to be more self-guiding in their studies than at pre-vocational schools. In general, students need to take full ownership for their studies and feel responsible for their study activities in vocational education. The majority of students that come from pre-vocational schools lack these skills to a higher or lesser degree (Severiens and Verstegen 2007).

To study self-regulated learning in pre-vocational education, researchers need instruments that have high reliability and validity. The present study examined, for the first time, the factorial validity and reliability of the Motivated Strategies for Learning Questionnaire (MSLQ) for students in pre-vocational secondary education, in order to see if we can accurately measure self-regulated learning of pre-vocational students in the Netherlands. Currently, there is insufficient evidence on the validity and reliability of the MSLQ for measuring self-regulated learning of students in pre-vocational secondary education. Next, we discuss, successively, the levels of Dutch secondary education, the components of the MSLQ and the focus of the current study.

### Levels of Dutch secondary education

In the Netherlands students start secondary education around the age of twelve, when they have finished primary education. Secondary education has three levels, of which pre-vocational education is considered the lowest track. Of all the students in secondary education the group in pre-vocational education is the largest; around 50% of the students in secondary education is in pre-vocational education (Peetsma and Van der Veen 2013; Prince 2014; Dutch Inspectorate of Education 2021). The other half of the population is in senior general secondary education or pre-university education. Pre-vocational education takes four years to complete and grands admission to vocational education and training, as well as to senior general secondary education. Pre-vocational education has three different educational tracks. The theoretical track, which is considered the highest track in pre-vocational education, offers only theoretical subjects and grands admission to the highest tracks in vocational education and training. The middle track, called the advanced vocational track, offers theoretical subjects, but on a lower level than the theoretical track. Students in the advanced vocational track are also offered vocational subjects in the senior years for a limited number of hours per week (Luyten et al., 2003; Prince 2014). The basic vocational track in pre-vocational education is regarded as the lowest track. In this track students choose a specialisation from the start in which they will be educated, for example technical education where they will be trained to be constructors or carpenters, or care & welfare education in which they will be trained to obtain an assisting position in health care.

Pre-vocational education, especially the lower tracks, has a negative image in the Netherlands; it is associated with learning and behavioural problems and unqualified teachers, and it is seen as of lower level than the other forms of secondary education (Van Daalen 2010; Prince 2014). This negative image, combined with limited abilities for transfer can negatively influence students’ self-image and decrease their perceptions of future opportunities (Peetsma and Van der Veen 2013). Students in pre-vocational education run a higher risk of being diagnosed with learning difficulties. They are also more often confronted with academic failure. Repeated negative perceptions of school performance may lead to a downward spiral of decreasing motivation and thus an increased risk of dropping out of school or loss of well-being while attending school (Minneart et al. 2017; Dickhäuser et al. 2011). This negative trend is reinforced when students do not have the ability to regulate their own motivation and learning behaviour (Dickhäuser et al. 2011). Self-regulation is seen as a pre-requisite for durable effectiveness in learning and motivation (Prince 2014). Despite concerns about the average achievement levels and decreasing motivation in Dutch pre-vocational education, it has not been comprehensively studied how self-regulated learning skills develop over time at this level of Dutch secondary education (Dutch Inspectorate of Education 2021). Insight in these developments are needed to design appropriate interventions and teaching approaches to support pre-vocational students in the development of self-regulated learning skills.

### Measuring self-regulated learning

The Motivated Strategies for Learning Questionnaire (MSLQ) is a commonly used instrument that is used world-wide to measure self-regulated learning (Dinsmore et al. 2008; Jackson 2018; Roth et al., 2016; Zimmerman 2008). Originally, the MSLQ was developed to measure self-regulated learning with college students in the USA. It was designed as a self-report questionnaire to measure the three elements in self-regulated learning: motivation, metacognition, and behaviour (Pintrich 2004; Pintrich et al. 1991, 1993; Wolters et al. 2005) for students in tertiary education, regardless of discipline (Soemantri et al. 2018). Below, we first explain the MSLQ in more detail. Next, we argue why the MSLQ may be suitable for measuring self-regulated learning among our target group of pre-vocational students.

The version of the MSLQ considered in this study has two main parts: a section on motivation and a section on learning strategies (Soemantri, 2018). Table 1 presents the MSLQ scales and subscales. The motivational section consists of three scales and six motivational subscales. The section on learning strategies consists of two scales and nine learning strategy subscales (Hilpert et al. 2013). It is generally accepted that self-regulated learning refers to the process in which students actively regulate their learning through cognitions, metacognitions, motivation and behaviour (Trautner and Schwinger 2018; Schunk & Zimmerman 1994; Zimmerman et al. 1992). The use of learning strategies is important for pre-vocational students, since they mainly have to learn from and during practical tasks (Jossberger, 2020). The MSLQ is a world-wide accepted questionnaire in which all these different aspects of self-regulated learning can be measured as well as the correlations between the different aspects of self-regulated learning, such as motivation and learning strategies (Artino 2005; Haelermans 2022).

**Table 1:** Sections, scales, and subscales of the MSLQ (Haelermans 2022)

Section	Scale	Subscale	Abbreviation subscale
Motivation	Value	1. Intrinsic goal orientation	IGO
		2. Extrinsic goal orientation	EGO
		3. Task value	TV
	Expectancy	4. Control of learning beliefs	CLB
		5. Self-efficacy for learning	SE
	Affect	6. Test anxiety	TA
Learning Strategies	Cognitive and Metacognitive Strategies	7. Rehearsal	R
		8. Elaboration	ELA
		9. Organization	ORG
		10. Critical thinking	CT
		11. Metacognitive self-regulation	MSR
	Resource Management	12. Time & study environment	TSE
		13. Effort regulation	ER
		14. Peer learning	PL
		15. Help seeking	HS

### Applications of the MSLQ

The MSLQ has been used by several researchers to examine the relationship between self-regulated learning and academic achievement at the college level (Al-Harthy et al. 2010; Jackson 2018; Kitsantas et al., 2008; Lynch 2006, 2010; Lynch and Trujillo 2011; Vanderstoep et al. 1996). All in all, these studies have found students’ self-efficacy scores to be the strongest predictors of course performance and of academic achievement. Also, students’ scores on the scales of effort regulation and time & study environment (management) have been shown to be the next significant predictors of achievement (Jackson 2018).

The MSLQ has been used to assess self-regulated learning for various student populations. We consider two examples. Mundia et al. (2021) performed a field survey to determine differences in MSLQ- scores determined by gender, age, educational level and school, the sample consisted of 310 secondary education students in Brunei. The researchers report statistically significant differences across all four participants’ variables for three scales (EGO, R, ER), where it was notable that on EGO all female participants scored higher than their male counterparts. Furthermore, females scored higher on six other scales (R, ER, TA, ORG, and CLB). Differences in age accounted for different scores in six scales (CT, TSE, IGO, EGO, R, and ER). For four subscales no significant differences were detected (SE, TV, ELA, PL). The researchers recommend further MSLQ-based studies that use more robust statistics such as CFA, as the present study does. Van der Veen and Peetsma (2009) used parts of the MSLQ, combined with other instruments for the collection of their data to investigate the decline in self-regulated learning behaviour of students in the first year of Dutch secondary education. They indeed found a decline in self-regulated learning behaviour, especially in the use of (metacognitive) learning strategies. Furthermore, a strong relationship was found between self-regulated learning behaviour and intrinsic value.

### Reliability and validity of the MSLQ

The reliability of the MSLQ has been tested by the developers (Pintrich et al., 1993). Furthermore, it has been demonstrated that the MSLQ can be used with various populations and in many different kinds of disciplines, with good confidence for obtaining generally reliable scores (e.g. Midgley et al. 1998; Eturan Ilker et al. 2014; Feiz et al., 2013; Haelermans 2022; Taylor 2012). The 15 scales of the MSLQ may be used together or separately (Pintrich et al. 1991); in many applications only one of the two major sections is used (Hilpert et al. 2013). The application of the MSLQ showed acceptable but varying estimated reliability in general higher education courses, represented with Cronbach alpha values, ranging from 0.41 to 0.78 for learning strategies scale and from 0.50 to 0.93 for motivation scale (Duncan and McKeachie 2005; Kosnin 2007; Soemantri et al. 2018). Pintrich and DeGroot (1990) described a five-factor structure when using data from junior high students. The five factors were labelled expectancy, value, affect, learning strategies, and self-regulation (Hilpert et al. 2013).

The validity of self-report questionnaires is influenced by the ability of participants in relating the questions in the questionnaire to their own learning experiences and thus to the analysis of their learning in a conscious manner (Jackson 2018; Roth et al. 2016). Therefore, research on the MSLQ with diverse populations and in diverse situation is needed (Cho and Summers 2012; Pintrich et al., 2000). Studies have shown problems with the MSLQ, for example, a lack of evidence for the factor structure associated with measuring self-regulated learning (Credé and Phillips 2011; Dunn et al. 2012; Hilpert et al. 2013; Jackson 2018; Muis et al. 2007; Tock and Moxley 2017). Factorial validity of the MSLQ has not been comprehensively studied for single administrations of the entire questionnaire (Hilpert et al. 2013). Few studies have examined all of the subscales in a single administration of the MSLQ, save for those conducted by the creators of the scale, Pintrich and DeGroot (1990), Pintrich et al. (1991) and Pintrich et al., (1993), and Rao and Sachs (1999). The scarcity of measurement work that examines the full factorial validity of the instrument is an important gap in the MSLQ literature (Hilpert et al. 2013). Furthermore, Pintrich et al. (2000) acknowledged problems of lack of a stronger fit between the theoretical model and empirical data and recommended more research on self-regulation and control instruments such as the MSLQ with diverse populations across a range of age and achievement levels (Jackson 2018; Pintrich et al. 2000; Wolters et al. 2005). Consequently, there is a need to assess the validity of the MSLQ for diverse groups in different context and environments (Jackson 2018).

### The current study

Self-regulated learning is a multidimensional complex construct (Boekaerts and Corno 2005; Schuitema et al. 2012). To study the various features of self-regulated learning in pre-vocational education, researchers need instruments that have high reliability and validity (Jackson 2018). The present study examined, for the first time, the factorial validity and reliability of the MSLQ for students in pre-vocational secondary education, in order to see if we can accurately measure self-regulated learning of pre-vocational students in the Netherlands. Currently, there is insufficient evidence on the validity and reliability of the MSLQ for measuring self-regulated learning of students in pre-vocational secondary education. For secondary education of European countries, we found that factorial validity of the MSLQ has only been investigated in the Turkish (Karadeniz et al., 2008) and Spanish context (Segura-Robles et al. 2021). However, since the Spanish and the Turkish educational contexts are quite different from the Dutch educational context, and these studies didn’t differentiate between levels of secondary education, we didn’t expect that the results of these studies would transfer to Dutch pre-vocational students. Furthermore, given the challenges and modifications presented in the literature, we expected that some modifications to the factorial models are required, but we didn’t have hypotheses for specific subscales.

## Methods

### Design

The research design is cross-sectional and non-experimental. The data were collected with an online version of the MSLQ. The research was carried out with due consideration to all relevant ethical issues and in line with BERA’s Ethical Guidelines for Educational Research.

### Sample

The sample consisted of 509 students in pre-vocational education. The students attended four secondary schools in the eastern part of the Netherlands. 46% of the students were girls. The pre-vocational students came from all three pre-vocational tracks. 59% were in the theoretical track, 31% were in the advanced vocational track, and 10% were in the basic vocational track. Furthermore, the pre-vocational students came from all 4 years of the educational programs. 22% of the students were in grade 7, 26% in grade 8, 17% were in grade 9, and 36% of the students were in grade 10.

### Variables

The MSLQ consists of two main sections: a motivational section with 31 items and a section on learning strategies with 50 items (see, also Table 1). The answer options of all 81 items are on a five-point Likert scale, with 1 = is (almost) never true for me, 2 = is sometimes true for me, 3 = neutral/do not understand the question, 4 = is often true for me, and 5 = is (almost) always true for me (Soemantri et al. 2018). Table 2 presents the six subscales of the motivational section, together with the estimated reliability scores found by Duncan and McKeachie (1991) for a sample of college students (*n* = 356 university; *n* = 24 community college).

**Table 2:** Number of items, example items and estimated reliability of the scales of the motivational section of the MSLQ

Scale	Number of items	Example question	α
Intrinsic Goal Orientation (IGO)	4	- The most satisfying thing for me in class is trying to understand the content as thoroughly as possible. - When I have an opportunity in this class, I choose course assignments that I can learn from even if they don’t guarantee a good grade.	0.74
Extrinsic Goal Orientation (EGO)	4	- Getting a good grade in this class is the most satisfying thing for me right now. - If I can, I want to get better grades in this class than most of the other students.	0.62
Task Value (TV)	6	- I have an uneasy, upset feeling when I take a test. - It is important for me to learn the course material in this class.	0.90
Control of Learning Beliefs (CLB)	4	- If I study in appropriate ways, then I will be able to learn the material in this course. - It’s my own fault if I don’t learn the material in this course.	0.68
Self-Efficacy for Learning and Performance (SE)	8	- I believe I will receive an excellent grade in this class. - I’m confident I can do an excellent job on the assignments and tests in this course.	0.93
Test Anxiety (TA)	5	- I am very interested in the content area of this course.	0.80

Table 3 presents the nine subscales of the section on learning strategies, together with the estimated reliability scores found by Duncan and McKeachie (1991) for a sample of college students (*n* = 356 university; *n* = 24 community college).

**Table 3:** Number of items, example items and estimated reliability of the scales of the learning strategies section of the MSLQ

Scale	Number of items	Example question	α
Rehearsal (R)	4	- When I study for this class, I practice saying the material to myself over and over. - I make lists of important terms for this course and memorize the list.	0.69
Elaboration (ELA)	6	- When reading for this class, I try to relate the material to what I already know. - I try to apply ideas from course readings in other class activities such as lecture and discussion.	0.76
Organization (ORG)	4	- When I study for this course, I outline the material to help me organize my thoughts. - I make simple charts, diagrams, or tables to help me organize course material.	0.64
Critical Thinking (CT)	5	- I often find myself questioning things I hear or read in this course to decide if I find them convincing. - I try to play around with ideas of my own related to what I am learning in this course.	0.80
Metacognitive Self-Regulation (MSR)	12	- Before I study new course material thoroughly, I often skim it to see how it is organized. - If course materials are difficult to understand, I change the way I read the material.	0.79
Time and Study Environment (TSE)	8	- I have a regular place set aside for studying. - I attend class regularly.	0.76
Effort Regulation (ER)	4	- I work hard in this class even if I don’t like what we are doing. - Even when course materials are dull and uninteresting, I manage to keep working until I finish.	0.69
Peer Learning (PL)	3	- When studying for this course, I often try to explain the material to a classmate or a friend. - I try to work with other students from this class to complete the course assignments.	0.76
Help Seeking (HS)	4	- When I can’t understand the material in this course, I ask another student in this class for help. - I try to identify students in this class whom I can ask for help if necessary.	0.52

### Procedure

There are multiple approaches for translating questionnaires and how to consider cultural adaptations (Epstein et al. 2015; Muñiz et al. 2013; Segura-Robles et al. 2021). For this study, the following steps were taken:

1. The first two authors, who are both fluently in English and native Dutch speakers, translated the questionnaire to Dutch.
2. Feedback on the translated questionnaire was provided by ten experienced teachers in pre-vocational education in the Netherlands. The feedback led to various modifications of the questionnaire (e.g., suggestions to use less complex words).
3. In a pilot study the translated and adapted MSLQ was administered to 115 pre-vocational students from a pre-vocational school in the east of the Netherlands, grades 9 and 10. The students in this pilot study did not participate in the current study. Using the results on reliability and the factor structures from the pilot study the questionnaire was further adapted, e.g., the wording was changed for some items (e.g., when an item loaded high on two different subscales).
4. After performing the back translation, the final questionnaire was considered very close to the original MSLQ.

The data collection began on October 15, 2021 and ended on November 30, 2021. The questionnaire was distributed online using Qualtrics. Students could use their mobile phones or another electronic device (e.g., Chromebook, iPad) to complete the questionnaire. The confidentiality and anonymity of participants and their data were guaranteed. The questionnaire was completed during homeroom classes under guidance of the students’ homeroom teachers. The students were given the opportunity to scan a QR code and complete the questionnaire on their phone or they could copy-paste a direct link to the questionnaire that was given to them through Google Classroom and allowed them to complete the questionnaire on their electronic device such as Chromebook or iPad.

### Statistical analysis plan

To explore the linear relationships between the observed items and subscales, and to check whether the Pearson correlations between items of a subscale are sufficiently high for further investigating the factor structure, correlations were calculated between the 31 motivation items and between the 50 items associated with the learning strategies. In psychological research, Cohen’s (1988) conventions to interpret effect size are commonly used. A correlation coefficient of 0.10, 0.30 or 0.50 or higher is thought to represent, respectively, a small, moderate and large correlation. Next, descriptive statistics were calculated for the MSLQ sections (Motivation and Learning Strategies) and associated subscales: mean, standard deviation, minimum and maximum. Moreover, the reliability of the sections and subscales were assessed using Cronbach’s alpha (Cronbach 1951). The correlations, descriptive statistics and reliability estimates were calculated with SPSS (version 26). The value of Cronbach’s alpha depends on the number of items associated with the mean subscale (Sijtsma 2009; Warrens 2015). Therefore, alpha values should be considered in light of the number of items associated with a subscale score (Hoekstra et al. 2019). A value of Cronbach’s alpha of 0.70 is generally considered to reflect an acceptable value of reliability (Cortina 1993) regardless of the number of items of the subscale.

Factorial validity of the components of the MSLQ was investigated with confirmatory factor analysis (CFA) using the statistical software program R (R Core Team, 2017) and the lavaan package (Roseel 2012). Factorial validity was first studied for each subscale separately (one-factor models), followed by second order factor models for the scales (see Table 1). Model parameters were estimated using diagonally weighted least squares (DWLS) estimation, which is suitable for ordered categories. To assess model fit we used the chi-squared statistic (χ2) and associated degrees of freedom, the root mean square error of approximation (RMSEA) and its 90% confidence interval (90% CI), the comparative fit index (CFI), the Tucker-Lewis Index (TLI), and the Standardized Root Mean Square Residual (SRMR). Given the large sample size (*N* = 594), it was likely that all χ2-based model tests would be significant (Hu and Bentler 1999). Thus, we focused primarily on the other fit measures. A cut-off point of ≤ 0.10 for RMSEA and ≥ 0.90 for both CFI and TLI indicated a moderate fit. The cut-off points for RMSEA at ≤ 0.06 and for CFI and TLI at ≥ 0.95 indicated a good fit. For the SRMR the cut-off point for a good fit was set at ≤ 0.08 (Hu and Bentler 1999). For individual subscales, if some statistic indicated that the associated model had only moderate model fit, modification indices were assessed to see what changes would improve model fit. Modifications were only applied if the changes were substantively meaningful. Modified scales are denoted with an asterisk (*).

## Results

We first checked whether the correlations between items of the same subscale were sufficiently high for further exploring the factor structure. Supplementary (Excel) files 1 and 2 present the correlation tables between, respectively, the 31 motivation items and the 50 items associated with the learning strategies. The items of the subscale IGO are numbered IGO1, IGO2, etc. The numbering of items of other subscales is analogous. Correlations between items of the same motivation subscales will be discussed first. There are strong correlations between IGO1 and IGO2 (0.60); all other correlations between the IGO items are weak (< 0.30). EGO1 has a weak correlation with EGO3 (0.19) and with EGO4 (0.20). Furthermore, EGO2 has a weak correlation with EGO4 (0.25). For the subscale TV it is item TV1 that has weak correlations with all the other items (0.14-0.25). For the subscale CLB it is CLB4 that has the weakest correlations, especially with CLB1, a coefficient of only 0.08 (with CLB2: 0.15; with CLB3: 0.13). The subscale SE shows moderate correlations between most items, ranging from 0.22 (SE5 and SE7) to 0.49 (SE1 and SE6). The subscale TA shows moderate to strong correlations, ranging from 0.37 (TA1 and TA3) to 0.70 (TA4 and TA5). For the learning strategies subscales, correlations between items of the same learning strategy are often below 0.30. Only six correlation coefficients are 0.30 or above and could therefore be considered moderate (0.37 for R2 and R4; 0.40 for ORG3 and ORG4; 0.38 for TSE3 and TSE7; 0.32 for TSE3 and TSE8; 0.32 for ER2 and ER4; 0.30 for HS3 and HS4). All other correlations between items associated with the same learning strategy were below 0.30 and were therefore considered weak. This means that multicollinearity is probably not an issue for the analyses. Furthermore, the moderate and strong correlations between items of the same subscales could be an indication that items have a shared component (e.g., they measure the same underlying latent variable). However, the weak correlations may indicate that some the estimated reliability of some subscales will be low. Overall, the correlations found do provide grounds to further investigate the factor structure.

Table 4 presents various descriptive statistics for the fifteen MSLQ subscale scores. The second column of Table 4 presents the number of items for each subscale. The third column of Table 4 presents the mean score, scored on a 5-point Likert scale, ranging from 1 (is almost never true for me) to 5 (is almost always true for me), with 3 as the neutral score (I don’t know/I have no opinion). As the third column shows, most mean scores are around 3.00, only EGO and CLB are above 3.60 and SE is above 3.50, meaning that, on average, these students valued their self-regulated learning skills as neutral to moderately positive.

**Table 4:** Descriptive statistics for the MSLQ and the sections and subscales of the MSLQ

Construct	#items	M	SD	Min	Max	α
MSLQ	81	3.24	0.31	1.75	4.15	0.863
Motivation	31	3.34	0.40	1.00	4.52	0.815
Learning strategies	50	3.18	0.38	1.46	4.44	0.854
IGO	4	3.03	0.68	2.47	4.03	0.501
EGO	4	3.62	0.69	3.08	3.98	0.599
TV	6	3.23	0.63	2.77	3.64	0.690
CLB	4	3.66	0.56	3.13	4.02	0.448
SE	8	3.57	0.57	3.14	3.72	0.790
TA	5	2.91	0.96	2.79	3.06	0.822
R	4	3.40	0.64	3.06	3.65	0.451
ELA	6	3.24	0.57	3.07	3.40	0.502
ORG	4	3.13	0.64	2.94	3.46	0.235
CT	5	3.03	0.62	2.72	3.21	0.511
MSR	12	3.07	0.46	2.92	3.48	0.600
TSE	8	3.29	0.45	2.92	3.62	0.316
ER	4	3.20	0.59	2.86	3.48	0.235
PL	3	3.08	0.71	2.78	3.40	0.237
HS	4	3.25	0.63	2.91	3.53	0.416

Note: #items denotes the number of items of the sections and subscales. IGO = Intrinsic goal orientation; EGO = Extrinsic goal orientation; TV = Task value; CLB = Control of learning beliefs; SE = Self efficacy; TA = Test anxiety; R = Rehearsal; ELA = Elaboration; ORG = Organisation; CT = Critical thinking; MSR = Metacognitive self-regulation; TSE = Time & study environment; ER = Effort regulation; PL = Peer learning; HS = Help seeking

The last column of Table 4 presents the values of Cronbach’s alpha. The values associated with the MSLQ and the sections Motivation and Learning Strategies are quite high (> 0.81) for our sample. This may in part be due to the large number of items (Warrens 2015; Hoekstra et al. 2019). The alpha values of the individual subscales vary widely. Acceptable values (> 0.70) were found only for the subscales SE and TA, both overall and for each group separately. The low estimated reliabilities of some subscales are in line with the weak correlations between the associated items. At this point, no items were removed from the subscales. The factorial validity of the subscales is first studied in the following.

Table 5 presents the fit indices for the CFA models for the six individual motivation subscales. The model fit is good for IGO, CLB and SE in terms of CFI (0.977-0.987), TLI (0.961-0.986) and SRMR (0.029-0.058). For IGO and SE the model fit is moderate in terms of RMSEA (0.063 and 0.085). For CLB the model fit is also good in terms of RMSEA (0.052). Furthermore, the model fit is poor for EGO, TV and TA in terms of RMSEA (> 0.11). For these three subscales, modification indices were assessed to see if the model fit could be improved.

**Table 5:** Goodness-of-fit indices and modifications for CFA models for motivational subscales

Subscale	Modification	χ2	df	CFI	TLI	RMSEA	90% CI for RMSEA	SRMR
IGO		5.98	2	0.995	0.986	0.063	[0.000,0.123]	0.035
EGO		14.64	2	0.977	0.930	0.112	[0.063,0.168]	0.049
EGO*	EGO1 ~ ~ EGO2	0.58	1	1.000	1.000	0.000	[0.000,0.106]	0.010
TV		85.84	9	0.944	0.906	0.130	[0.106.155]	0.075
TV*	-TV3	29.1	5	0.970	0.940	0.097	[065,0.133]	0.053
CLB		4.75	2	0.987	0.961	0.052	[0.000,0.114]	0.029
SE		94.09	20	0.979	0.970	0.085	[0.068,0.103]	0.058
TA		38.07	5	0.991	0.982	0.114	[0.082,0.149]	0.048
TA*	TA4 ~ ~ TA5	5.73	4	1.000	0.999	0.290	[0.000,0.078]	0.021
TA**	TA2 ~ ~ TA3	2.02	3	1.000	1.000	0.000	[0.000,0.064]	0.013

Note: IGO = Intrinsic goal orientation; EGO = Extrinsic goal orientation; TV = Task value; CLB = Control of learning beliefs; SE = Self efficacy; TA = Test anxiety

After adding a correlation between EGO1 and EGO2 to the factor model, the model fit of EGO became excellent (CFI = TLI = 1.00, RMSEA = 0.00, SRMR = 0.010). A substantive justification for this modification is that EGO1 and EGO2 are about students’ own grades, whereas EGO3 and EGO4 are about students’ grades in relation to other students.

The model fit provides empirical support that extrinsic goal orientation can be assessed among pre-vocational students with these four items. However, students seem to make a distinction between the content of EGO1 and EGO2, on the one hand, and EGO3 and EGO4, on the other hand. See also the discussion of correlations between items.

Removing TV3 gave a moderate model fit for subscale TV (CFI = 0.970, TLI = 0.940, RMSEA = 0.097, SRMR = 0.053). The content of TV3 seems to be different from that of the other items of TV: it appeals to intrinsic motivation, whereas the other TV items consider extrinsic motivational processes. Moreover, an excellent model fit (CFI = TLI = 1.00, RMSEA = 0.00, SRMR = 0.013) for TA was obtained after adding correlation between TA4 and TA5, as well as a correlation between TA2 and TA3. Substantively, these modifications make sense: items TA4 and TA5 consider emotions while making tests, whereas TA2 and TA3 consider cognitive processes while making tests.

Table 6 presents the correlations between the MSLQ subscales. The values of the correlations between the motivation subscales present a mixed view. Some motivation subscales are moderately correlated (e.g., IGO, EGO and TV), but the correlations between other motivation subscales are rather weak (e.g., TV and CLB with TA). The correlations between the learning strategies are in many cases moderate to high (e.g., MR, ELA, CT and MSR).

**Table 6:** Correlations between MSLQ subscales

	IGO	EGO	TV	CLB	SE	TA	*R*	ELA	ORG	CT	MSR	TSE	ER	PL
IGO	1													
EGO	0.364	1												
TV	0.424	0.466	1											
CLB	0.161	0.212	0.202	1										
SE	0.313	0.423	0.494	0.318	1									
TA	0.172	0.263	0.070	− 0.015	− 0.277	1								
R	0.140	0.098	0.126	0.047	0.011	0.129	1							
ELA	0.118	0.132	0.043	− 0.007	0.011	0.138	0.570	1						
ORG	0.113	0.104	0.137	− 0.020	− 0.021	0.225	0.454	0.460	1					
CT	0.195	0.121	0.010	0.010	0.014	0.215	0.369	0.481	0.268	1				
MSR	0.121	0.123	0.143	0.040	0.065	0.172	0.432	0.630	0.454	0.499	1			
TSE	0.149	0.097	0.053	0.020	0.096	0.113	0.431	0.441	0.299	0.382	0.384	1		
ER	− 0.093	− 0.023	− 0.045	0.000	0.005	0.102	0.190	0.271	0.169	0.298	0.417	0.268	1	
PL	0.152	0.084	0.106	0.003	0.042	0.141	0.274	0.330	0.255	0.295	0.338	0.256	0.207	1
HS	0.065	0.066	0.040	0.111	0.081	0.127	0.311	0.402	0.218	0.290	0.437	0.318	0.331	0.309

Table 7 presents the fit indices for the CFA models for the nine learning strategy subscales. The model fit is good for R, ORG, CT, PL and HS in terms of all four fit indices (CFI > 0.98, TLI > 0.95, RMSEA < 0.054 and SRMR < 0.040). Furthermore, the model fit is poor for ELA, MSR, TSE and ER in terms of at least one of the fit indices. For these four subscales, modification indices were assessed to see if the model fit can be improved.

**Table 7:** Goodness-of-fit indices and modifications for CFA models for learning strategies subscales

Subscale	Modifications	χ2	df	CFI	TLI	RMSEA	90% CI for RMSEA	SRMR
R		0.37	2	1.000	1.000	0.000	[0.000,0.051]	0.009
ELA		39.16	9	0.915	0.859	0.081	[0.056,0.108]	0.054
ELA*	-ELA4	8.976	5	0.985	0.970	0.040	[0.000,0.081]	0.031
ORG		4.39	2	0.984	0.953	0.049	[0.000,0.112]	0.030
CT		12.13	5	0.984	0.967	0.053	[0.014,0.092]	0.039
MSR		162.09	54	0.909	0.889	0.063	[0.052,0.074]	0.060
MSR*	-MSR9	92.044	44	0.953	0.941	0.046	[0.033,0.060]	0.049
MSR**	-MSR3&9	42.086	35	0.991	0.989	0.020	[0.039,0.050]	0.037
TSE		199.09	20	0.674	0.544	0.133	[0.116,0.150]	0.097
TSE*	-TSE5	73.321	14	0.851	0.776	0.091	[0.071,0.112]	0.069
TSE**	-TSE4&5	26.257	9	0.950	0.917	0.061	[0.035,0.089]	0.049
ER		7.30	2	0.939	0.818	0.072	[0.021,0.132]	0.040
ER*	-ER3	0.00	0	1.000	1.000	0.000	[0.000,0.000]	0.000
PL		0.00	0	1.000	1.000	0.000	[0.000,0.000]	0.000
HS		1.61	2	1.000	1.000	0.000	[0.000,0.082]	0.019

Note: R = Rehearsal; ELA = Elaboration; ORG = Organisation; CT = Critical thinking; MSR = Metacognitive self-regulation; TSE = Time & study environment; ER = Effort regulation; PL = Peer learning; HS = Help seeking

Removing ELA4 gave a good model fit for subscale ELA (CFI = 0.985, TLI = 0.970, RMSEA = 0.097, SRMR = 0.031). A substantive justification for this modification is that ELA4 is about making a summary when studying for tests, which could be viewed by pre-vocational students as preparation for study and not as studying itself. Furthermore, a good model fit (CFI = 0.991, TLI = 0.989, RMSEA = 0.020, SRMR = 0.037) was obtained for MSR after removing both MSR3 and MSR9. An explanation for the misfit of MSR3 and MSR9 is that both items contain a negation in the first part of the question (MSR3: “If there is something I don’t understand while studying, I go back and try to find out what it is about”; MSR9: “I do not start reading, but I first think about a subject and about what it is I need to know about it”), which is not the case for the other items of the subscale. The negation may lead to a different interpretation of these questions by pre-vocational students. Furthermore, removing both TSE4 and TSE5 only led to a moderate model fit in terms of TLI (0.917) for the subscale TSE. The wording of item TSE5 may be not specific enough for pre-vocational students, because it contains words like study and make, in general and for most subjects (“I learn and make my homework in general for most subjects”).

Finally, an excellent model fit (CFI = TLI = 1.00, RMSEA = SRMR = 0.00) was obtained for ER after removing ER3. ER3 exhibits a low correlation with ER2 (0.033) and with ER4 (0.053). Furthermore, unlike the other ER items, item ER3 contains a conditional clause and two options (ER3: “If the materials are too difficult for me, I stop studying or I only study those parts that I understand (or make the exercises that I do understand in case of assignments”) which may lead to interpretation difficulties for pre-vocational students. However, after removing ER3 the model for EL is exactly identified, since there are only three EL items remaining. In this case, factorial validity is difficult to establish.

Next, we consider the second order factor models for the five scales (see Table 1). Table 8 presents the fit indices for the CFA models corresponding to the five scales Value, Expectancy, Affect, Cognitive and Metacognitive strategies and Resource Management. The scale Expectancy had a good fit in terms of indices CFI (0.961), TLI (0.950) and SRMR (0.069) and a moderate fit in terms of RMSEA (0.085). Furthermore, the scale Affect had a rather good fit in terms of indices CFI (0.991), TLI (0.977) and SRMR (0.048), but a poor fit in terms of RMSEA (0.129). The other three scales had a poor model fit in terms of all four indices (CFI < 0.83; TLI < 0.81; RMSEA > 0.10 and SRMR > 0.09).

**Table 8:** Goodness-of-fit indices and modifications of the CFA models for the five scales

Scale	X^2^	df	CFI	TLI	RMSEA	90% CI for RMSEA	SRMR
Value	1117.87	74	0.809	0.766	0.167	[0.158,0.176]	0.132
Expectancy	243.62	52	0.961	0.950	0.085	[0.075,0.096]	0.069
Affect	38.07	4	0.991	0.977	0.129	[0.094,0.168]	0.048
CM Strategies	2713.34	429	0.822	0.807	0.102	[0.099,0.106]	0.091
RM	1129.33	171	0.711	0.666	0.114	[0.108,0.121]	0.097

Note: CM Strategies = Cognitive and Metacognitive Strategies; RM = Resource Management

Modification indices were assessed to see if the model fit of some of the scales Value, Affect, Cognitive and Metacognitive strategies and Resource Management presented in Table 8 could be improved. However, for each of the scales at least seven modifications were required to obtain a moderate model fit in terms of all statistics. Moreover, unlike the modifications applied to the subscales in Tables 5 and 7, most modifications were difficult to justify substantively (e.g. factor loadings of item to other subscales than their original subscale), and are therefore not presented. In addition, we explored whether (performance) differences between the educational levels contributed to poor fit of the second order models for four of the five scales. As a first step towards multiple group analyses, we considered separate factor models for the theoretical track (*n* = 299) and the advanced vocational track (*n* = 157). The number of basic vocational students (*n* = 53) was not high enough for these additional analyses. Table 9 presents the fit indices for the CFA models corresponding to the five scales, split for the theoretical track and advanced vocational track students. The model fit for the scale Value is poor in terms of all indices for both the theoretical track and advanced vocational track students, as well as for the complete sample (Table 8). Furthermore, for the scale Affect, the model fit for both the theoretical track and advanced vocational students is rather good in terms of indices CFI, TLI and SRMR, but a poor fit in terms of RMSEA, which is very similar to the model fit for the complete sample. Hence, the poor model fit for the scales Value and Affect (in terms of RMSEA) may not be due to differences between the educational tracks. For the scales Expectancy, Cognitive and Metacognitive strategies and Resource Management the model fit could not be computed for the advanced vocational students since the estimation procedure for these scales did not converge. Since, for all three scales, the model fit statistics for the theoretical track students are quite similar to the model fit statistics for the complete sample, the model fit may not be dependent on (performance) differences between the educational tracks. The non-convergence of some of the models presented in Table 9 seem to be related to the smaller sample (e.g., the sample of advanced vocational track students only).

**Table 9:** Goodness-of-fit indices and modifications of the CFA models for the five scales, split for theoretical track and advanced vocational track students

Scale	X^2^	df	CFI	TLI	RMSEA	90% CI for RMSEA	SRMR
Theoretical track (*n* = 299)							
Value	1052.84	74	0.739	0.679	0.211	[0.200,0.222]	0.167
Expectancy	190.65	52	0.935	0.918	0.095	[0.080,0.109]	0.081
Affect	22.28	4	0.993	0.982	0.124	[0.077,0.176]	0.047
CM Strategies	1934.88	429	0.801	0.784	0.109	[0.104,0.113]	0.102
RM	696.057	148	0.751	0.712	0.111	[0.103,0.120]	0.101
Advanced vocational track (*n* = 157)							
Value	174.29	74	0.940	0.926	0.093	[0.075,0.111]	0.089
Expectancy	No convergence						
Affect	18.14	4	0.987	0.968	0.151	[0.085,0.224]	0.065
CM Strategies	No convergence						
RM	No convergence						

Note: CM Strategies = Cognitive and Metacognitive Strategies; RM = Resource Management

Based on the poor model fit for the three scales Value, Cognitive and Metacognitive strategies and Resource Management, we recommend against the use of scores based on these scales. Furthermore, we considered the three order factor models for the two sections Motivation and Learning Strategies (see Table 1). As can be expected from the results on the two factor models for the five scales, both sections had a poor model fit in terms of all four indices. In addition, modification indices were assessed to see if the model fit of some of the sections could be improved. For both sections, a lot of modifications were required to obtain a moderate model fit in terms of all statistics, and most modifications were difficult to justify substantively. The results are not presented here, and we recommend against the use of scores based on the two sections.

## Discussion

Self-regulated learning is a multidimensional complex construct (Boekaerts and Corno 2005; Schuitema et al. 2012). To study the various features of self-regulated learning in pre-vocational education, researchers need instruments that have high reliability and validity. The MSLQ is an instrument that has been used worldwide for measuring self-regulated learning. However, the reliability and factorial validity of the MSLQ has not been assessed for the context of pre-vocational education. Furthermore, in European secondary education factorial validity of the MSLQ has only been investigated in a Turkish (Karadeniz et al., 2008) and Spanish context (Segura-Robles et al. 2021). The reliability and factorial validity of the MSLQ has not been studied for students in Dutch secondary education. Moreover, since the Spanish and the Turkish educational contexts are quite different from the Dutch educational context, and these studies didn’t differentiate between levels of secondary education, we didn’t expect that the results of these studies would transfer to Dutch pre-vocational students. Therefore, the aim of this study was to investigate, for the first time, the factorial validity and reliability of the MSLQ for pre-vocational students using a Dutch sample.

### Summary of the results

Reliability was calculated for the total score of the MSLQ, for the sum scores associated with both sections (Motivation and Learning strategies) and for each subscale separately, using Cronbach’s alpha (Cronbach 1951). The value of Cronbach’s alpha associated with the MSLQ total score indicated high reliability (0.86). Furthermore, both sections also exhibited high reliability (Motivation: 0.82; Learning strategies: 0.85). However, the reliability of the subscales was generally much lower. We only found an alpha value of > 0.70 for the sum score associated with two subscales, namely SE and TA, where a value of 0.70 indicates an acceptable value of reliability (Cortina 1993). All other subscales had values of Cronbach’s alpha < 0.70, with extreme values for ORG and ER, which both had an alpha value of. 235. Thus, in terms of Cronbach’s alpha we found evidence of high reliability for the MSLQ total score, the sum scores based on the section and the sum scores associated with subscale SE and TA. However, we didn’t find evidence for high reliability for the other subscales. These findings seem closely related to the dependence of Cronbach’s value on the number of items on which a score is based (Hoekstra et al. 2019; Sijtsma 2009; Warrens 2015). The total score of the MSLQ and the sum scores corresponding to the two sections are based on much more items that the individual subscales of the MSLQ.

We found evidence of high factor validity for most of the MSLQ subscales. In other words, the constructs measured with these subscales can be assessed quite well among Dutch pre-vocational students. For some subscales modifications to the original models were required. The model fit was improved by either, removing an item from a subscale, adding a correlation between two items, or adding a factor loading between an item and a construct. For all subscales, each modification could be justified substantively, following the content of the items. We obtained good model fit for the motivation subscales IGO, EGO, CLB and TA. Furthermore, we obtained moderate fit for the subscales TV (after modifications) and SE. In addition, we obtained good model fit for the learning strategies scale subscales R, ELA, CT, and MSR. Moreover, we obtained moderate model fit for the subscale ORG. We could not provide evidence for a good or moderate model fit for the subscale TSE. In other words, all MSLQ subscales, except the TSE scale, can be used to assess the corresponding MSLQ component among Dutch pre-vocational students in a valid way. The scale Expectancy overall had a good model fit, and the scale Affect overall had a moderate fit. The other three scales and the factor model associated with the two sections Motivation and Learning Strategies had a poor model fit.

### Implications for practice

The model fit indices indicate that we can validly and reliably assess the first order MSLQ components among Dutch pre-vocational students. However, before some subscales are used it is recommended that a specific item of the scale is omitted, to have high factorial validity (see Tables 5 and 7): omit item 3 for the subscale Task Value, item 4 for subscale the subscale Elaboration, items 3 and 9 for the subscale Metacognitive self-regulation, items 4 and 5 for the subscale Time & study environment, and item 3 for the subscale Effort regulation.

Furthermore, the model fit indices indicate that we can validly and reliably assess one second order MSLQ component among Dutch pre-vocational students, namely the scale Expectancy. The poor model fit for the other second factor models (for scales) and the two third order factor models for the sections indicates that substantial improvements of the items in some of the subscales are needed to improve validity of these measurements. Scores based on the four scales Value, Affect, Cognitive and Metacognitive strategies and Resource Management, the two sections Motivation and Learning Strategies, or the complete MSLQ, should not be used.

### Implications for research and limitations

The results on factorial validity presented in this study disagree to some extent with results reported on factorial validity of MSLQ in a different context and/or with secondary school students. In a research project in Turkey, among 1,114 students in the age of 12–18 years, the main problems were with items in the SE subscale. For that reason, the researchers removed several items from the SE subscale as well as item EGO3 (Karadeniz et al., 2008), whereas we have not done any modification to SE and in EGO we added a correlation between EGO1 and EGO2 instead. In a Spanish study, researchers found IGO3 to be invalid and placed it in subscale EGO, which provided a good model fit in a research project with 307 secondary school students in Spain Segura-Robles et al. 2021). In contrast, in our study IGO3 did not cause problems in terms of factorial validity. In Jacksons’ (2018) validation study of the MSLQ among STEM students from non-privileged background, only 48 items of the original 81 were left in the final model. In this model the whole subscales Extrinsic goal orientation, Test anxiety, Peer learning and Help seeking were left out and also half of the subscale Metacognitive self-regulation from the original model. In contrast, in the current study we only removed seven items (TV3, ELA4, MSR3, MSR9, TSE4, TSE5 and ER3) to obtain a good or moderate model fit.

The sample used in this study consists of four secondary schools in the eastern part of the Netherland. Since the sample did not include secondary schools from other regions of the Netherlands, the sample may not be representative for the population of Dutch pre-vocational students. Future studies on reliability and factorial validity could include schools from other regions, both urban and rural, of the Netherlands. Since still little is known about how self-regulated learning skills develop in Dutch pre-vocational education (Dutch Inspectorate of Education 2021), it is important to investigate these phenomena. Future studies may also consider other self-report methods for measuring self-regulated learning, like the Learning and Study Strategies Inventory (Weinstein & Palmer, 2002) and the Metacognitive Awareness Inventory (Schraw and Dennison 1994). The psychometric properties of these latter instruments have not been comprehensively studied with regard to Dutch pre-vocational student populations.

The past decades, many pre-vocational schools in the Netherlands have developed innovative learning programs that aim to enhance self-regulated learning of students (Oostdam et al., 2006; Schuitema et al. 2012; Stroet et al., 2016). To evaluate these developments and programs we need more insight into how self-regulated learning skills can be validly and reliably measured for this student population. Furthermore, in addition to pre-vocational education, future studies could include the higher educational levels of secondary schools, namely senior general education, which prepares students for higher professional education, and pre-university education, which prepares students for university.

### Conclusions

To study self-regulated learning in pre-vocational education, researchers need instruments that have high reliability and validity. This study investigated, for the first time, the factorial validity and reliability of the MSLQ for students in pre-vocational education in the Netherlands. For all MSLQ subscales, evidence was found that the constructs measured with these subscales can be assessed quite well among Dutch pre-vocational students. Certain subscales require that a specific item is removed to have high factorial validity. Furthermore, the modifications required for some of the factor models provided insight in the way certain items of the MSLQ are interpreted by pre-vocational students in the Netherlands. We found no strong evidence that the constructs measured with the MSLQ sections Motivation and Learning Strategies and the four scales Value, Affect, Cognitive and Metacognitive strategies and Resource Management can be assessed well among Dutch pre-vocational students. Therefore, scores based on the complete MSLQ, the two sections, or the four scales should not be used.

## Electronic supplementary material

Below is the link to the electronic supplementary material.

Supplementary Material 1

Supplementary Material 2

## Data Availability

The datasets used and analysed for the current study are available from the corresponding author on reasonable request. The R code used for the analyses of the current study are available from the corresponding author on reasonable request.

## Abbreviations

BERA: British Educational Research Association
CFA: confirmatory factor analysis
CFI: comparative fit index
CI: confidence interval
CLB: subscale Control of learning beliefs
CT: subscale Critical thinking
DWLS: diagonally weighted least squares (estimation)
EGO: subscale Extrinsic goal orientation
ELA: subscale Elaboration
ER: subscale Effort regulation
HAVO: senior general secondary education
HS: subscale Help seeking
IGO: subscale Intrinsic goal orientation
MSLQ: Motivated Strategies for Learning Questionnaire
MSR: subscale Metacognitive self-regulation
ORG: subscale Organization
PL: subscale Peer learning
R: subscale Rehearsal
RMSEA: root mean square error of approximation
SE: subscale Self-efficacy for learning
SRMR: Standardized Root Mean Square Residual TA-subscale Test anxiety
TLI: Tucker-Lewis index
TSE: subscale Time & study environment
TV: subscale Task value
VWO: pre-university education
